# *Denopelopia amicitia*, a new Tanypodinae from Brazil (Diptera, Chironomidae)

**DOI:** 10.3897/zookeys.553.5988

**Published:** 2016-01-14

**Authors:** Galileu P.S. Dantas, Neusa Hamada, Humberto F. Mendes

**Affiliations:** 1 Instituto Nacional de Pesquisas da Amazônia, Caixa Postal 478, 69011-970, Manaus, AM, Brazil; 2 Universidade Federal de Alfenas, Instituto de Ciências da Natureza, Rua Gabriel da Silva Monteiro, 700, Centro, 32130-000, Alfenas – MG, Brazil

**Keywords:** Tanypodinae, Pentaneurini, Amazonian, Neotropical Region, taxonomy, aquatic Insects

## Abstract

A new species of *Denopelopia* from Brazil is described based on adult male and pupa. The male of the new species can be distinguished from all other species of the genus by the genitalia and fore-tibial spur morphology. The pupa is very similar to those of *Denopelopia atria*, but it can be distinguished by the absence of distinct constrictions in the respiratory atrium of the thoracic horn. Generic diagnosis to male and pupa of *Denopelopia* is emended and keys to male and pupae of known species are provided.

## Introduction

The tanypod genus *Denopelopia* was erected by Roback and Rutter (1988) based on a single species (*Denopelopia atria*) from the Nearctic region. According to these authors, *Denopelopia* is closely related to *Telmatopelopia* Fittkau and *Zavrelimyia* Fittkau, these suppositions were recently corroborated by Silva and Ekrem (2015).

After the genus description, one undescribed species was reported from Panama based on adult male (Spies and Reiss 1996). So far, *Denopelopia* is composed of five described species, three of them with Asian distribution; Cheng and Wang (2005) described *Denopelopia diaoluonica*, *Denopelopia bractea* and *Denopelopia viridula*, from China, Kobayashi and Endo (2008) transferred *Yaequintus irioquereus* Sasa & Suzuki to the genus *Denopelopia* as senior synonym of *Denopelopia bractea*. The last species described on the genus, *Denopelopia moema*, was collected in Midwestern Brazil (Silva et al. 2014). Only one species, *Denopelopia atria*, has immature stages described, the remaining are known only as adult males.

In the present study, a new species from the Brazilian Amazon rainforest is described based on adult male and pupa, the generic diagnosis to male and pupa of *Denopelopia* (Roback and Rutter 1988, Murray and Fittkau 1989) is emended and keys to males and pupae are provided.

## Materials and methods

A pupa was collected in a small pond using a hand net. It was reared in laboratory isolated in a vial to obtain the associated adult; for further details on rearing techniques see Mendes (2002). The material examined was slide-mounted in Euparal, following the procedures outlined by Pinder (1986, 1989). The colour is described as observed in specimen conserved in alcohol. The general terminology follows Sæther (1980). The holotype of the named species was deposited in the Invertebrates collections of the Instituto Nacional de Pesquisas da Amazônia (INPA), Amazonas, Manaus, Brazil.

## Taxonomy

### Denopelopia

Taxon classificationAnimaliaDipteraChironomidae

Roback & Rutter, 1988

Denopelopia Roback & Rutter, 1988: 117.

#### Type species.

*Denopelopia atria* Roback & Rutter, 1988.

#### Emended diagnosis.

Based on the additional characters found in *Denopelopia amicitia* sp. n., the generic diagnosis for the pupa and male of *Denopelopia* given by Roback and Rutter (1988) and Murray and Fittkau (1989) must be emended. **Male**: scutal tubercle absent; wing densely covered with macrotrichia, costa not produced beyond R_4+5_, ending clearly before M_1+2_; tibial spurs with elongate apical tooth, more than half the length of the entire spur; tergite IX straight or rounded, with a transverse row of setae; gonocoxite with or without internal lobe. **Pupa**: wholly brown; thoracic horn elongated, with an apical nipple, a small plastron plate and respiratory atrium with or without constrictions; thoracic comb present; TVII with 3 lateral filaments; anal lobe with spines on the outer margin only; genital sac not surpassing apex of anal lobe.

### Denopelopia amicitia sp. n.

Taxon classificationAnimaliaDipteraChironomidae

http://zoobank.org/09A94352-BDD3-423A-A61B-1740C1452F0C

#### Type material.

Holotype male with pupal exuviae, Brazil, Amazonas State, Presidente Figueiredo, pisciculture pond, BR 174-Km 121, 01°55’50.2’’ S, 60°03’02.0” W, 10/xii/2012, G.P.S. Dantas, (INPA).

#### Diagnosis.

Male: AR 1.92; wing with well-developed anal lobe; spur of the fore tibia with the most basal tooth longer and slender than the other lateral teeth and strongly curved backwards; tergite IX rounded; gonocoxite with a well-developed setose lobe at the base. Pupa: with a distinct apical nipple, 38 µm long and 42 µm wide, L/W 0.9; absence of distinct constrictions in the respiratory atrium of the thoracic horn.

#### Etymology.

From Latin, *amicitia*, meaning friends, referring to friends who helped during fieldwork.

#### Male

(n = 1). Total length 2.90 mm. Wing length 1.7 mm. Total length/wing length 1.67. Wing length/length of profemur 2.34.

*General coloration* brown. Head yellow, occipital area brown; maxillary palp yellow; pedicel yellow, brown near the insertion of the flagellum; flagellomere I–XII light brown, VIII–VIV yellow. Thorax dark brown, pleura light brown. Legs yellow. Wings with membrane transparent, veins yellow. Abdomen: T I yellow, with light brown pigmentation on the anterolateral margin, T II–III yellow with clear brown band close to anterior margin; TIV with 1/3 anterior brown; TV–VIII brown, genitalia yellow.

**Figure 1.**
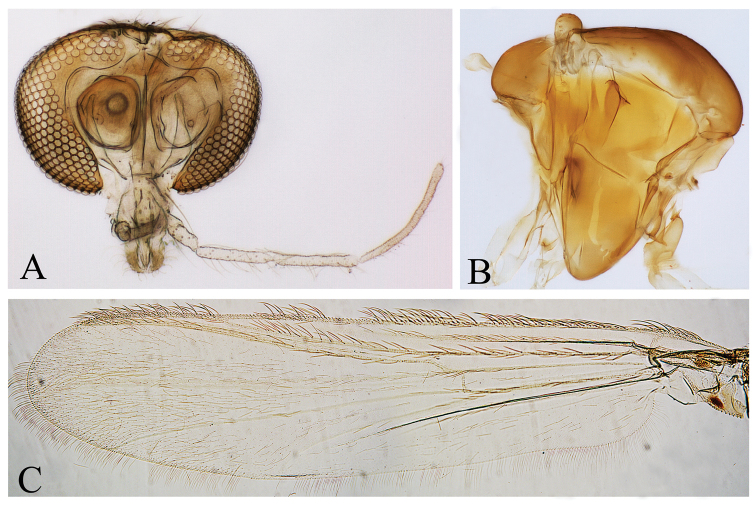
*Denopelopia amicitia* sp. n. Adult male: **A** head **B** thorax **C** wing.

*Head* (Fig. 1A). AR 1.92. Antenna with 14 flagellomere; thirteenth flagellomere 585 µm long. Apical flagellomere 92 µm long; 23 µm wide at base; with a subapical setae 65 µm long. Temporal setae 14. Clypeus 99 µm long, 82 µm wide, with 19 setae. Cibarial pump with anterior margin concave, 215 µm long and with orifice 95 from apex. Tentorium 165 µm long. First palpomere reduced. Palpomere lengths (1–5 in µm): 30; 65; 156; 168; 265.

*Thorax* (Fig. 1B). Scutal tubercle absent. Acrostichals 30, biserial, starting close to antepronotum and reaching half of scutum; dorsocentrals 14, biserial anteriorly and uniserial posteriorly; prealars 6, in a single irregular row; supraalar 1. Antepronotum with 3 setae. Scutellum with 22 setae, in three rows. Postnotum without setae.

**Figure 2.**
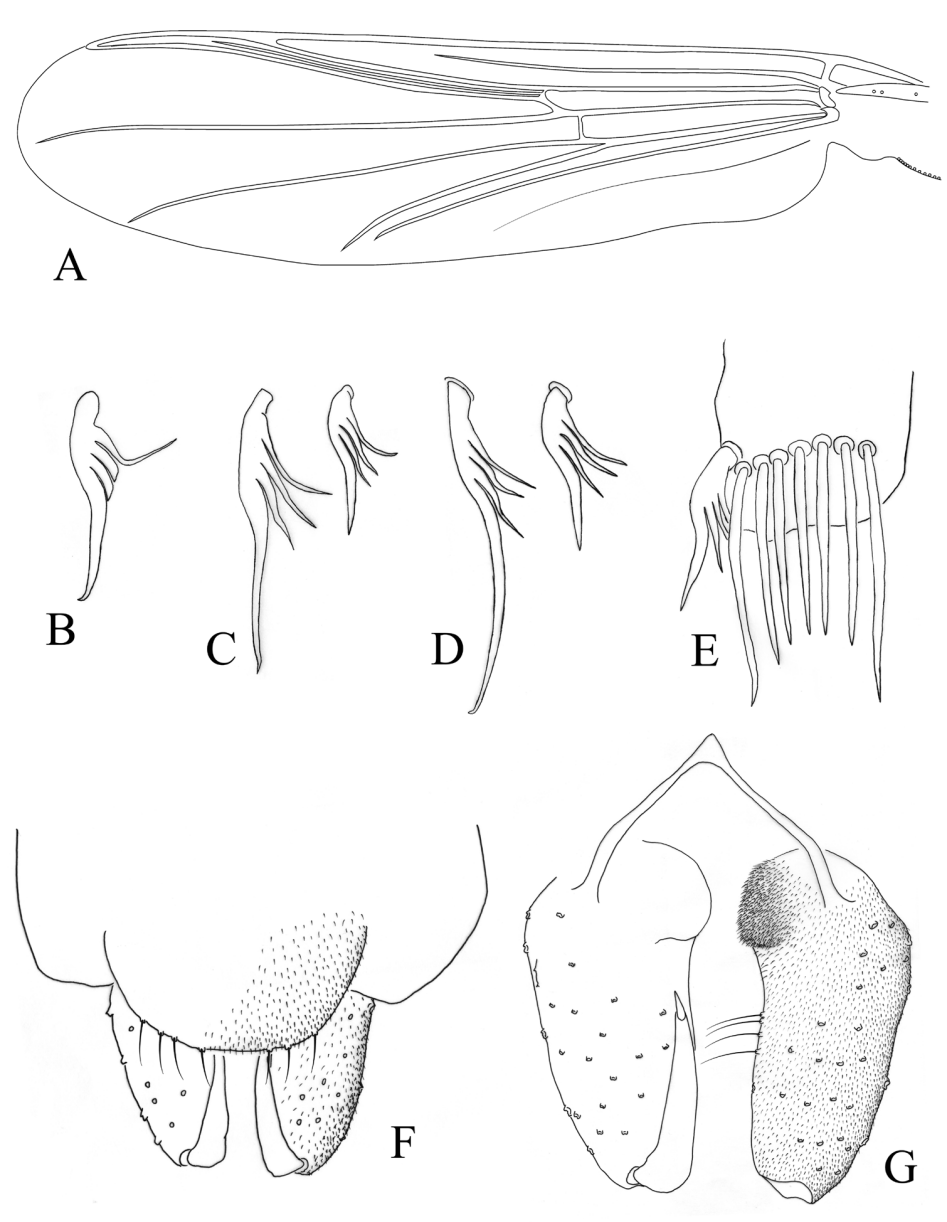
*Denopelopia amicitia* sp. n. Adult male: **A** wing **B** fore tibial spur **C** mid tibial spur **D** hind tibial spur **E** hypopygium in dorsal view **F** hypopygium with tergite IX removed.

*Wing* (Figs 1D, 2A). 1.7 mm long, 0.4 mm wide. VR 0.91 (Cu = 510 µm, M = 560 µm). Membrane with covering of macrotrichia, denser at the 1/3 distal; costa 1.6 mm long, not produced beyond apex of R_4+5_. R_2+3_ present. MCu proximal to the RM. Brachiolum with 3 setae. Squama with 12 setae. Anal lobe well-developed.

*Legs*. Fore leg: tibia with an apical, pectinate spur, 47 µm long, with three lateral teeth and one elongate apical tooth, the most basal tooth is longer and slender than the other lateral teeth and strongly curved backwards (Fig. 2B); two preapical setae 138 µm long; width at apex of tibia 40 µm; ta_1_ with one preapical stout setae, 86 µm long; ta_2_ with one preapical stout setae, 75 µm long; ta_3_ with two preapical stout setae, 72 µm long. Mid leg: tibia with two apical, pectinate spurs, 33 and 59 µm long, longest spur with three lateral teeth and one elongate apical tooth, shortest spur with three lateral teeth and one apical tooth (Fig. 2C); two preapical setae 119 µm long; width at apex of tibia 38 µm; ta_1_ with a preapical stout setae, 71 µm long; ta_2_ with two preapical stout setae, 65 µm long; ta_3_ with two preapical stout setae, 48 µm long. Hind leg: tibia with two apical, pectinate spurs, 39 and 78 µm long, longest spur with three lateral teeth and one elongate apical tooth, shortest spur with three lateral teeth and one apical tooth (Fig. 2D); one preapical setae 182 µm long; width at apex of tibia 44 µm; ta_1_ with one preapical stout setae, 90 µm long; ta_2_ with two preapical stout setae, 61 µm long. Tibial comb on hind leg with 7 bristles, the lateral longer than the medial ones (Fig. 2E). Claws of all legs normal, curved, sharply pointed; pulvilli absent. Lengths (in µm) and proportions of leg segments as in Table 1.

**Table 1.** Lengths (in µm) and proportions of leg segments in *Denopelopia amicitia* sp. n., male (n = 1).

	**Fe**	**ti**	**ta_1_**	**ta_2_**	**ta_3_**	**ta_4_**	**ta_5_**	**LR**	**BV**	**SV**
**p_1_**	729	850	743	422	341	204	109	0.87	2.16	2.12
**p_2_**	717	1016	758	412	294	184	109	0.75	2.49	2.29
**p_3_**	782	797	550	315	220	132	93	0.80	2.80	2.87

*Hypopygium* (Figs 2F-G). Tergite IX rounded, with 7 posterior setae. Anal point conical. Phallapodeme indistinct. Sternapodeme with triangular anterior process. Gonocoxite subcylindrical, 130 µm long, 40, 55, 65 µm wide at apex, at mid and at base respectively; with an internal setose lobe at the base, as in figure 2G. Gonostylus simple and slightly curved, 70 µm long; megaseta 10 µm long. HR 1.83; HV 4.10.

#### Pupa

(n = 1). Dimensions. Male abdomen 2.63 mm long.

**Figure 3.**
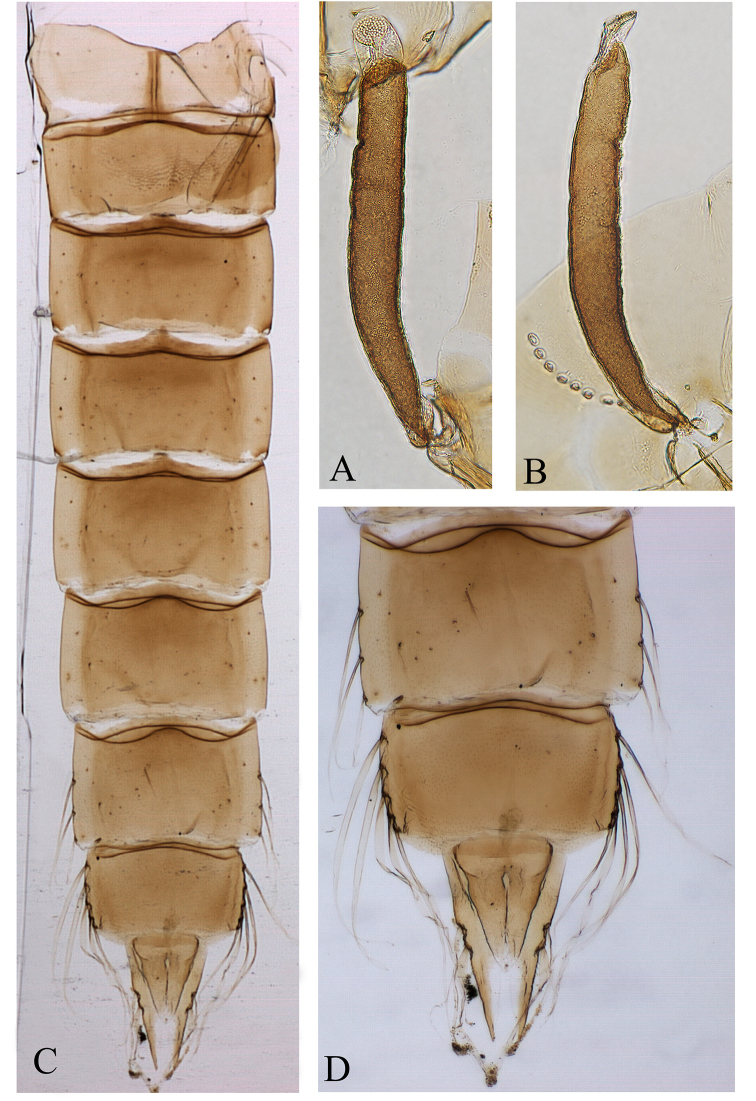
*Denopelopia amicitia* sp. n. Pupa: **A** thoracic horn, in frontal view **B** thoracic horn, in lateral view **C** abdomen, in dorsal view **D** T VII–VIII and Anal lobe.

*Coloration*. Cephalothorax brownish; thoracic horn dark brown, apical nipple transparent, plastron plate yellowish. Tergite I light brown, scar brown, T II-AL brown (Fig. 3C).

**Figure 4.**
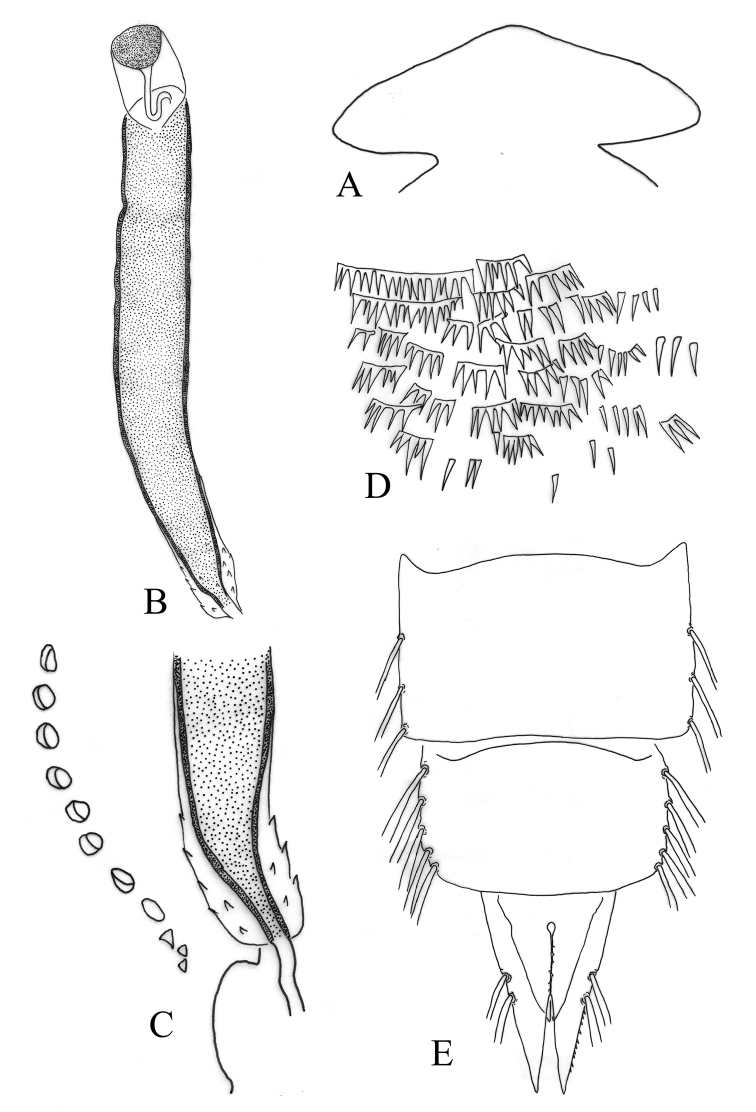
*Denopelopia amicitia* sp. n. Pupa: **A** frontal apotome **B** thoracic horn **C** base of thoracic horn, basal lobe and thoracic comb **D** shagreen of sternite II **E** T VII–VIII and Anal lobe.

*Cephalothorax*. Frontal apotome somewhat triangular (Fig. 4A). Wing sheath smooth, 1.1 mm long and 0.4 mm wide. Thoracic horn elongate and narrow (Fig. 3A-B, 4B-C), 390 µm long and 48 µm wide; with a distinct apical nipple, 38 µm long and 42 µm wide; plastron plate rounded, 28 µm long; aeropyle tube bended at the base, 53 µm long. Horn sac tubular, filling the respiratory atrium, except at the base; respiratory atrium without distinct constriction (Fig. 3A-B, 4B). Thoracic horn with small surface spines at the base (Fig. 4C). Basal lobe well developed, as in figure 4C. Thoracic comb with 12 conical teeth.

*Abdomen* (Fig. 3C). Tergite I without shagreen, T II–TVIII with shagreen composed by scattered fine spinules. Sternite II with a large field of shagreen composed by spinules arranged in combs (Fig. 4D). T I with a distinct and elongate scar, 170 µm long. T VII with 3 lateral filaments, 252 µm long; filaments placed at 132, 210 and 275 µm from base to apex of segment. T VIII with 5 lateral filaments, 372 µm long. Anal lobe as in figures 3D and 4E, 350 µm long, 213 µm wide at base and with two lateral macrosetae with sticky sheaths; outer margins with 12 spinules; inner margins without spinules. Genital sac smaller than anal lobe, 233 µm long, 162 µm wide at base. GS/AL 0.67.

### Systematic remarks

To date, *Labrundinia* is the only genus in the Tanypodinae which features a rounded male tergite IX, considered a synapomorphy for the group (Silva et al. 2015). The presence of this trait in *Denopelopia amicitia* n. sp. might suggest that a new genus should be erected to accommodate this species. However, considering that many tanypod genera are not properly studied, with only few described species, in many of these descriptions, this trait might have been overlooked by peers. Moreover, the pupa of *Denopelopia amicitia* sp. n. is very similar to that of *Denopelopia atria*, which leaves no doubt that the two species are congeneric. The adults of *Denopelopia* can be easily distinguished from those of *Labrundinia* by morphology of tibial spurs. In *Denopelopia* the spurs are well-developed with an elongated apical tooth and two spurs are present in the posterior tibia, while in *Labrundinia* the spurs are small, with subequal teeth and are absent in the posterior tibia. According to Roback and Rutter (1988), *Denopelopia* is closely related to *Telmatopelopia* and *Zavrelimyia* based on the overall morphology of adults and immature stages. However, adults of *Denopelopia* possess costa (C) not produced beyond R_4+5_ and ending clearly before M_1+2_, while in *Telmatopelopia* and *Zavrelimyia* costa is slightly produced and ends above or slightly beyond M_1+2_. The pupae of *Denopelopia* has anal macrosetae with adhesive sheath and spines only at the outer margin of the anal lobe, which contrast to *Telmatopelopia* with no adhesive sheath and *Zavrelimyia* that possesses spines both in the outer and inner margins of the anal lobe. In addition, *Denopelopia* has spines restricted to the base of the external membrane of the thoracic horn and possesses three lateral filaments in tergite VII, in contrast to *Telmatopelopia* and *Zavrelimyia*, which have the external membranes of the thoracic horn covered with spines and possess four lateral filaments in tergite VII. The larva of *Denopelopia* can be recognized by the presence of a trifid paraligula and elongated labial vesicles, in contrast to *Telmatopelopia* and *Zavrelimyia* where the paraligula is bifid and the labial vesicles are more or less rounded.

The male of *Denopelopia amicitia* sp. n. can be distinguished from all other species of the genus by the hypopygium morphology. It has a well-developed setose lobe at the base of the gonocoxite, which is absent or reduced in other species of the genus. In addition, the male of *Denopelopia amicitia* sp. n. has the anal lobe of the wing well-developed, which sets it apart from *Denopelopia viridula* and *Denopelopia diaoluonica*, that have it reduced and absent, respectively; the absence of two scale-shaped bristles at the apex of the anterior tibia, the well-developed anal lobe of the wing and the sternapodeme with a pointed anterior process, distinguishes *Denopelopia amicitia* sp. n. from *Denopelopia irioquerea*; the morphology of the anterior tibial spur and the rounded tergite IX distinguishes *Denopelopia amicitia* sp. n. from *Denopelopia moema* and *Denopelopia atria*. The pupal stage is similar to that of *Denopelopia atria* due to the morphology of the thoracic horn and the number of lateral filaments on tergite VII, but can be distinguished by the absence of distinct constrictions in the respiratory atrium and by the low length/width ratio of the apical nipple.

### Ecological notes

There is little information on the biology of *Denopelopia*, since only *Denopelopia atria* has its immature stages described. This species was described based on material collected and reared from a shallow drainage ditch amongst *Typha* sp., with low dissolved oxygen and relatively high iron concentrations (Roback and Rutter 1988) in Florida (USA). This species has also been collected in lentic environments in Costa Rica (Epler 2001) and an unreared larva of the genus was recorded from Southeastern Brazil by Trivinho-Strixino and Strixino (1995).

The pupa of *Denopelopia amicitia* sp. n. was collected in a small disabled pisciculture pond, associated with marginal vegetation, in a eutrophic environment where a carcass of a large animal, in advanced stages of decomposition, was observed.

Several attempts to collect additional material were made; however, the pond where it was collected was drained and sampling in the adjacent areas, such as streams and wetlands, was not successful.

### Key to adult males of *Denopelopia* (adapted from Cheng and Wang (2005) to include the Brazilian species)

**Table:** 

1	Apex of fore tibia with two large scale-like setae	***Denopelopia irioquerea* (Sasa & Suzuki, 2000)**
–	Fore tibiae without scale-like setae	**2**
2	Gonocoxite with a well-developed setose lobe at the base	***Denopelopia amicitia* sp. n**
–	Gonocoxite without a well-developed setose lobe at the base	**3**
3	Wing with well-developed anal lobe	**4**
–	Wing with reduced or absent anal lobe	**5**
4	Anterior margin of abdominal segment I and IV with distinctive brown spots	***Denopelopia moema* Silva, Wiedenbrug & Oliveira, 2014**
–	Anterior margin of abdominal segment I and IV without brown spots	***Denopelopia atria* Roback & Rutter, 1988**
5	AR > 0.9; wing with anal lobe reduced	***Denopelopia viridula* Cheng & Wang, 2005**
–	AR < 0.6; wing with anal lobe absent	***Denopelopia diaoluonica* Cheng & Wang, 2005**

### Key to pupae of *Denopelopia*

**Table:** 

1	Respiratory atrium without distinct constriction (Fig. 3A-B, 4C); proportion L/W of apical nipple of thoracic horn 0.9	***Denopelopia amicitia* sp. n**
–	Respiratory atrium with distinct constriction; proportion L/W of apical nipple of thoracic horn about 2.0	***Denopelopia atria* Roback & Rutter, 1988**

## Supplementary Material

XML Treatment for Denopelopia

XML Treatment for Denopelopia amicitia
